# SCOPE (Scapular Corrections for Posterior Escape) for posterior glenohumeral instability: technique of posterior acromial corrective osteotomy with optional glenoid corrective osteotomy

**DOI:** 10.1016/j.xrrt.2026.100852

**Published:** 2026-08-14

**Authors:** Jean-David Werthel, Matthias A. Zumstein, Bastian Sigrist, Bettina Hochreiter, Justine Fleurette, Beat Kaspar Moor, Gabriele Cirigliano, Christian Gerber

**Affiliations:** a Department of Orthopaedic Surgery, Hôpital Ambroise Paré, Boulogne-Billancourt, France; b Shoulder, Elbow and Orthopaedic Sports Medicine, Sonnenhof Orthopaedics, Bern, Switzerland; c Faculty of Medicine, University of Bern, Bern, Switzerland; d Faculty of Medicine, Health and Human Sciences, Macquarie University, Sydney, NSW, Australia; e Center for 3D Preoperative Planning and 3D Printing, Balgrist University Hospital, University of Zurich, Zurich, Switzerland; f Department of Orthopaedics, Balgrist University Hospital, University of Zurich, Zurich, Switzerland; g Department of Orthopaedic Surgery and Traumatology, Valais Hospital, Martigny, Switzerland

**Keywords:** Shoulder, Posterior glenohumeral instability, SCOPE, Scapinelli, Acromion, Posterior subluxation, PABB, PACO

Posterior instability is a complex entity that differs fundamentally from anterior instability.^4^ Whereas anterior instability typically follows a distinct traumatic event resulting in an anterior dislocation, a traumatic posterior dislocation is rarely the presenting event in posterior instability.^4^ Recurrent posterior instability is more commonly related to repetitive microtrauma^28^^,^^30^^,^^36^^,^^37^ with a wide spectrum of symptoms,^21^ ranging from activity-related pain to constant pain,^4^^,^^38^ subjective apprehension, and episodes of posterior subluxation of variable frequency, occasionally requiring reduction.^38^ Posterior instability may present with a static posterior glenohumeral subluxation, either with or without recurrent dynamic subluxation. When isolated, static posterior subluxation may only become apparent with the development of early (eccentric) glenohumeral osteoarthritis.^8^ Within this heterogeneous clinical presentation, the distinction between dynamic and static posterior instability associated with early-onset glenohumeral osteoarthritis remains incompletely understood.^8^ Numerous treatment strategies have been proposed for these patients, including physical therapy,^7^^,^^15^, ^16^, ^17^ a shoulder pacemaker,^25^^,^^26^ posterior capsulolabral repair,^1^^,^^24^^,^^31^^,^^40^ posterior bone-block procedures,^6^^,^^22^^,^^34^ and reverse Hill-Sachs remplissage.^19^ Although these approaches have distinct indications, high failure rates have been reported with each,^27^^,^^35^ and none directly addresses the abnormal scapular morphology increasingly recognized across static, dynamic, and combined forms of posterior instability.

More recently, patients with dynamic posterior instability and those with primary glenohumeral osteoarthritis associated with static posterior humeral head subluxation have been shown to share similar scapular morphologic features that differ from those of healthy shoulders.^2^^,^^3^^,^^20^ Specifically, these shoulders demonstrate a posterior acromion that is higher, shorter, and flatter,^2^^,^^3^^,^^20^ allowing greater posterior translation of the humeral head during anteroposterior loading (eg, as in forward elevation of the arm). In cadaveric biomechanical studies, these abnormal posterior translations have repeatedly been shown to be reducible by surgical corrections of posterior acromial morphology.^12^, ^13^, ^14^

These observations led to the development of the SCOPE (Scapular Corrections for Posterior Escape) approach. Because the pattern of scapular deformity varies among patients, correction is tailored to the underlying morphology and to the specific parameters requiring correction. Accordingly, posterior acromial bone block (PABB or modified Scapinelli), glenoid corrective osteotomy (GCO), and the novel posterior acromial corrective osteotomy (PACO) may be performed as standalone procedures or combined within the same reconstruction, depending on the deformity pattern. Given the encouraging preliminary results reported for PACO,^9^^,^^10^ the purpose of the present study is to describe the 3-dimensional pre-operative planning and to provide a detailed step-by-step surgical technique, including technical pearls and pitfalls.

## Surgical procedure

### Indications

The current clinical indication for the SCOPE approach is symptomatic posterior humeral head escape, presenting as static, dynamic, or combined posterior instability, that persists despite appropriate nonoperative treatment and is associated with abnormal glenoid and/or posterior acromial morphology in the absence of advanced glenohumeral osteoarthritis.

The practical morphologic reference values currently used in our planning process are based on the normal scapular measurements reported by Beeler et al^3^ and correspond approximately to one standard deviation beyond the mean normal value in the direction associated with posterior instability. Abnormal morphology is currently considered to include glenoid retroversion greater than 10°, glenoid inclination below 84°, posterior acromial coverage (PAC) below 70°, posterior acromial height (PAH) above 20 mm, critical shoulder angle (CSA) below 28°, and sagittal acromial tilt below 63°. In a patient fulfilling the clinical indications described above, the presence of at least one abnormal acromial parameter or at least one abnormal glenoid parameter supports consideration of the corresponding correction. These values are used as practical reference guides rather than as standalone operative thresholds. The authors expect that, with increasing experience and the availability of larger clinical series, these indications and procedure-selection criteria will be further refined.

### Pre-operative planning

The SCOPE approach is based on experimental work documenting that biomechanically an isolated correction of a single parameter is insufficient to restore posterior stability; rather, a combined correction of the relevant pathologic scapular parameters may be required. In particular, correcting excessive glenoid retroversion alone may not restore stability, and correcting posterior acromial morphology alone may also be insufficient when other major morphologic deviations persist. Accordingly, pre-operative three-dimensional (3D) planning aims to identify, and address, all biomechanically relevant deviations from normal scapular morphology.

Planning is based on a pre-operative computed tomography (CT) scan, which includes the entire scapula. The scapula is segmented and reconstructed in 3 dimensions, and a standardized set of morphologic measurements is obtained, including glenoid version, glenoid inclination, PAC, PAH, sagittal acromial tilt, and the CSA.^41^ These measurements, together with the three-dimensional scapular reconstruction, are compared with a statistical shape model (SSM) of a healthy scapula derived from 40 asymptomatic healthy shoulders^3^ (Fig. 1).

**Figure 1:**
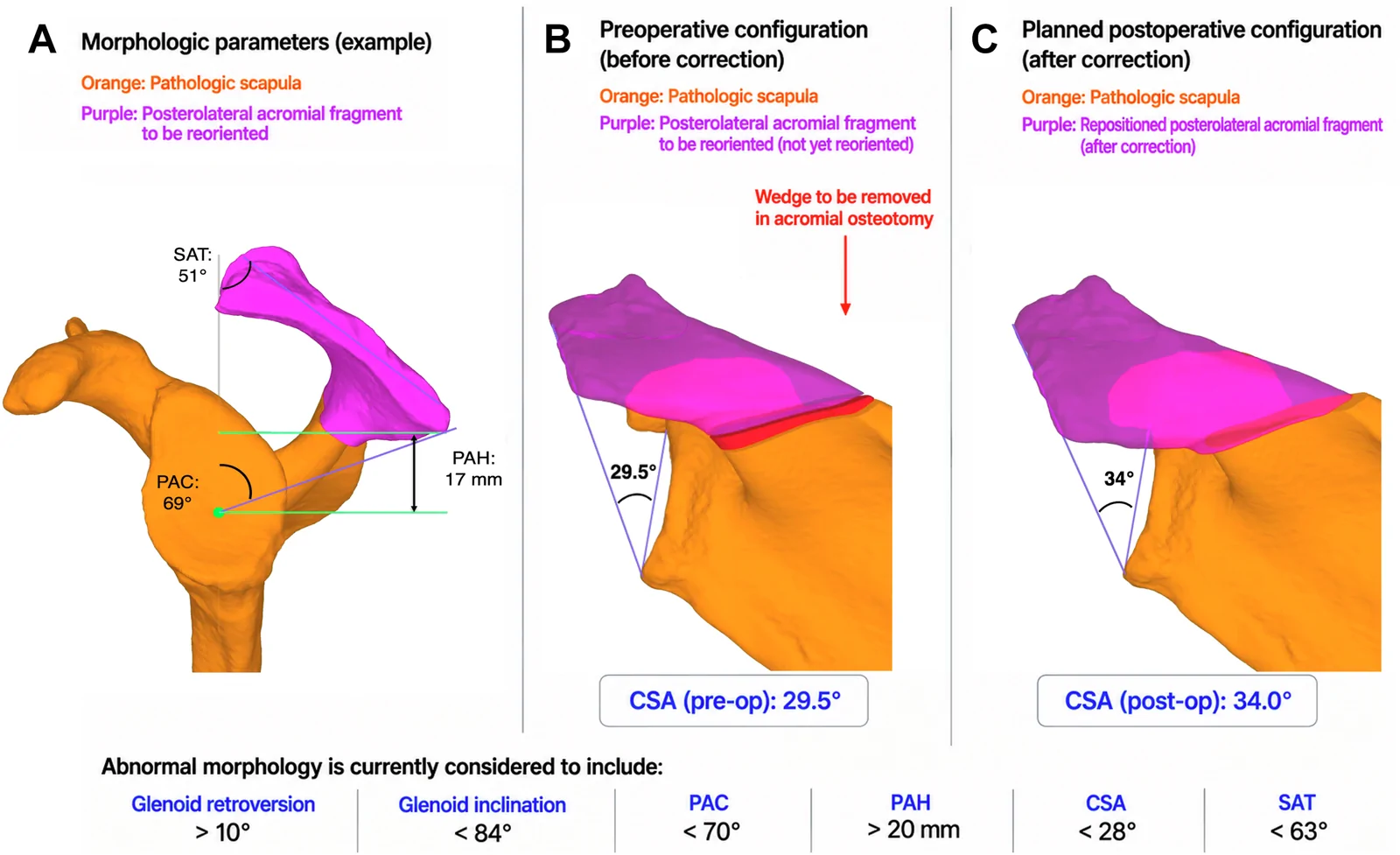
Quantitative three-dimensional assessment and simulation used for SCOPE planning. (**A**) Patient-specific pathologic scapula shown in orange. The posterolateral acromial fragment intended for reorientation is shown in purple. Posterior acromial coverage (PAC), posterior acromial height (PAH), and sagittal acromial tilt (SAT) are measured on the three-dimensional reconstruction to identify abnormal parameters and define the correction target. (**B**) Pre-operative configuration before acromial correction. The red region represents the patient-specific bone wedge planned for removal during the posterior acromial corrective osteotomy. The pre-operative critical shoulder angle (CSA) is shown. (**C**) Planned post-operative configuration after wedge removal and reorientation of the posterolateral acromial fragment. The planned translation and rotation of the fragment restore posterior acromial morphology and modify the CSA toward the intended target. The practical morphologic reference values currently used to guide planning are glenoid retroversion >10°, glenoid inclination <84°, PAC <70°, PAH >20 mm, CSA <28°, and SAT <63°. *SCOPE*, Scapular Corrections for Posterior Escape.

If the posterior acromion is found to be short, high, and/or flat, an acromial correction is planned. If the glenoid demonstrates excessive retroversion, a posterior GCO is additionally planned. Within the SCOPE armamentarium, 3 acromial procedures may be considered: PACO, a PABB, or a combination of the 2. The choice between these techniques is guided by the correction required to reproduce physiologic scapular morphology, as determined by comparison with the SSM (Fig. 1). The SSM-based analysis quantifies deviations in PAH, PAC, and lateral acromial extension, as well as glenoid version and inclination; the surgical plan is then tailored to correct the dominant abnormalities using PABB, PACO, posterior GCO, or a combination when necessary. Therefore, PABB is considered when the principal abnormality is insufficient posterior acromial length or coverage with preserved lateral extension. PACO is preferred when simultaneous correction of posterior height, posterior coverage, and lateral extension is required, particularly in the setting of a low CSA or marked external rotation of the acromion. GCO is considered when excessive retroversion and/or abnormal inclination is judged to contribute substantially to posterior humeral head escape (Fig. 2).

**Figure 2:**
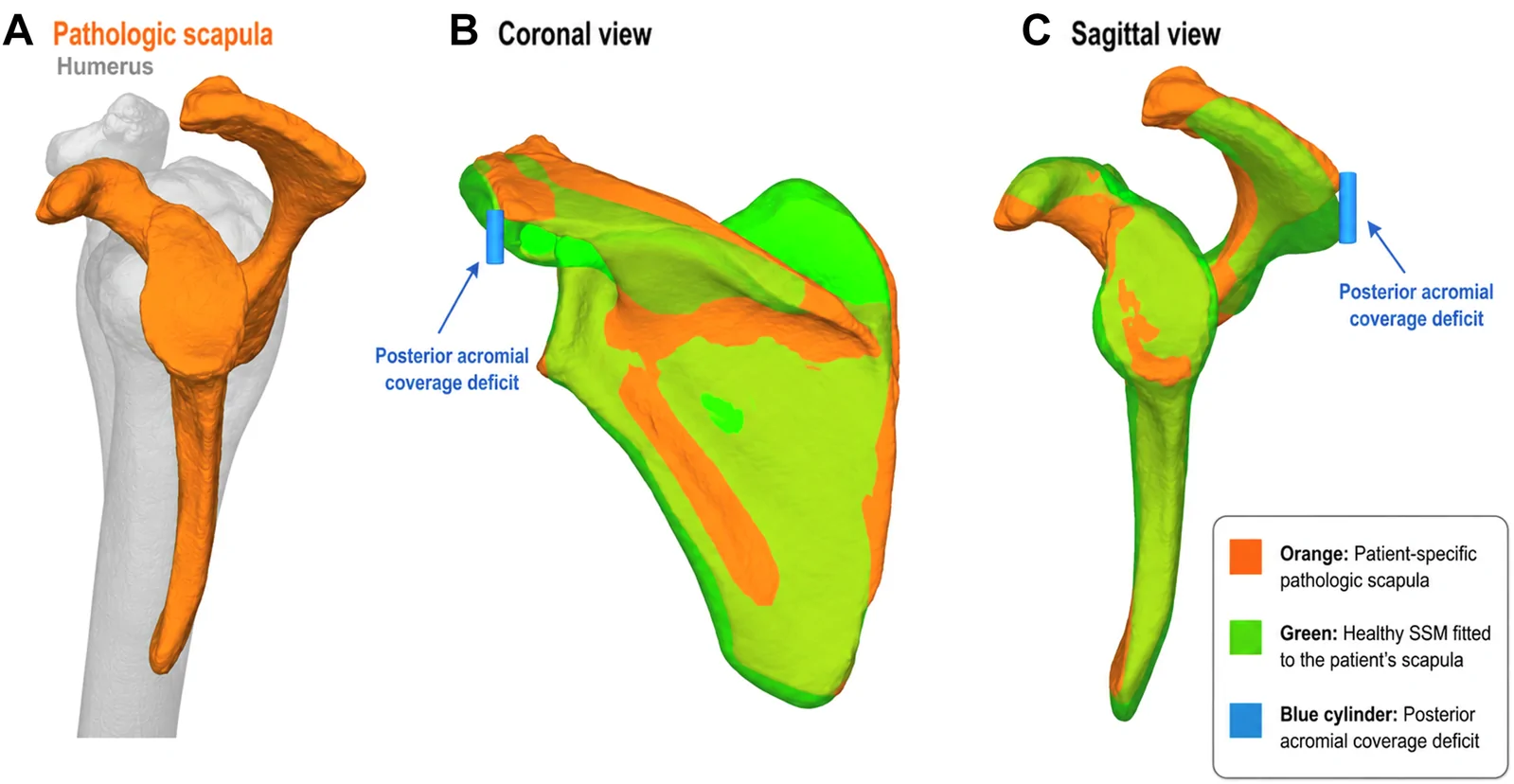
Patient-specific three-dimensional planning based on comparison with a healthy statistical shape model. (**A**) Patient-specific pathologic scapula shown in orange, with the humerus shown in gray. (**B** and **C**) Superimposition of the pathologic scapula *(orange)* and a healthy statistical shape model (SSM) fitted to the patient's scapula *(green)*. The blue cylinder highlights the posterolateral region in which the pathologic acromion fails to provide the posterior coverage predicted by the fitted healthy model. These comparisons identify the location and direction of the morphologic deviation and are used to define the correction target and select the appropriate SCOPE procedure or combination of procedures. *SCOPE*, Scapular Corrections for Posterior Escape.

A PABB lengthens the posterior acromion and may be sufficient in selected cases. However, PACO should be preferred in several situations, including: (1) a low CSA; (2) marked external rotation of the acromion, resulting in posterolateral deficiency despite a near-normal CSA (Fig. 3); and (3) insufficient lateral acromial extension to achieve adequate PAC with a PABB, or when a concomitant posterior glenoid opening-wedge osteotomy lateralizes the humeral head such that adequate PAC can no longer be achieved with a PABB. In such cases, a PABB alone may remain too medial to provide adequate posterior restraint and would not address the altered glenohumeral loading associated with a low CSA. By contrast, PACO enables simultaneous correction of posterior height, posterior coverage, and lateral extension and is intended to provide posterior osseous support through midrange motion rather than only at end-range elevation or abduction.

**Figure 3:**
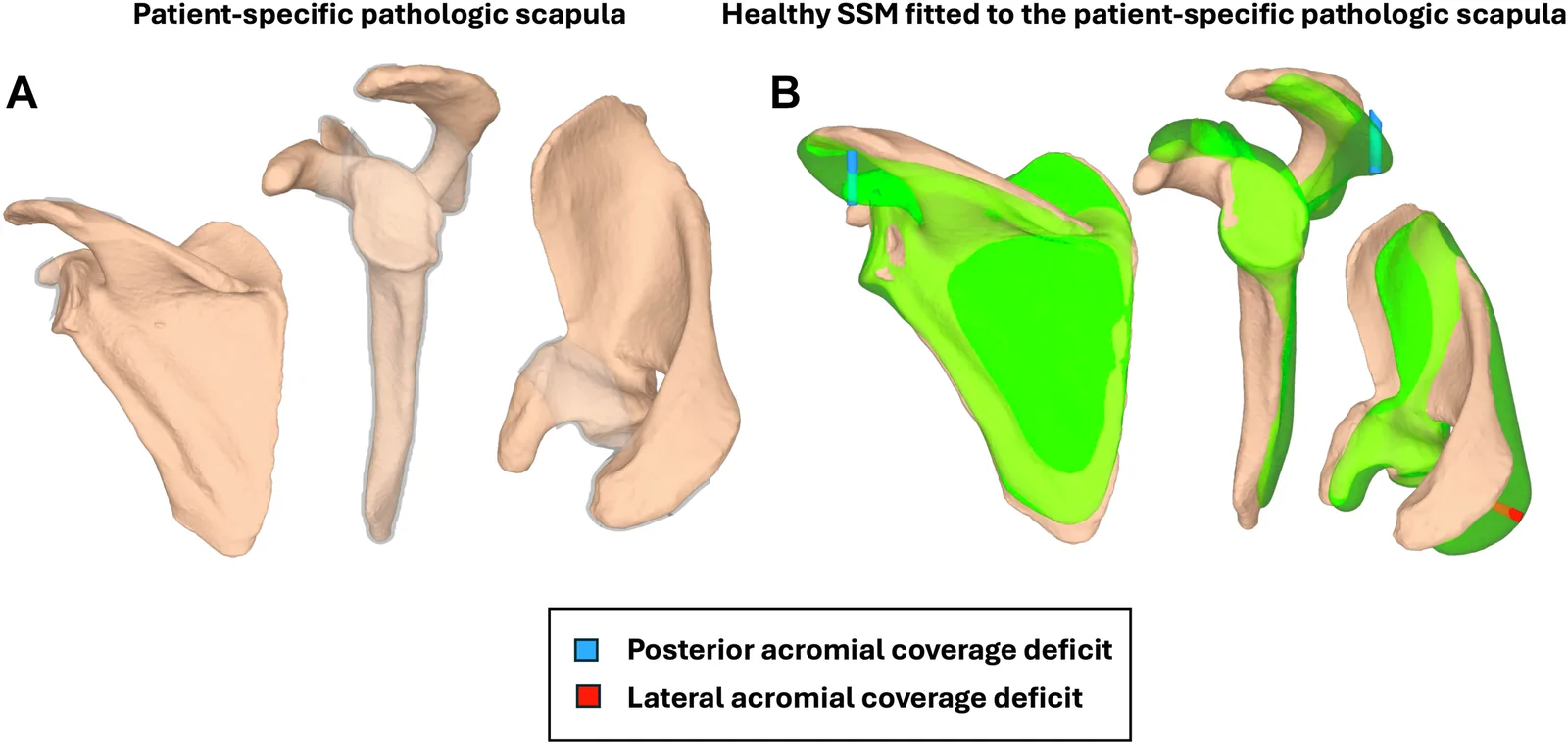
Example of an indication favoring posterior acromial corrective osteotomy (PACO). (**A**) Patient-specific pathologic scapula shown in three representative views. (**B**) Superimposition of the patient-specific pathologic scapula with a healthy statistical shape model (SSM) fitted to the patient's anatomy and shown in green. Blue markers indicate posterior acromial coverage deficiency, whereas the red marker indicates marked lateral acromial coverage deficiency. This pattern illustrates marked external rotation of the acromion with posterolateral deficiency despite a normal critical shoulder angle (PAC: 41.7°; PAH: 36.2 mm; SAT: 80.2°; CSA: 34°). *PAC*, posterior acromial coverage; *PAH*, posterior acromial height; *SAT*, sagittal acromial tilt; *CSA*, critical shoulder angle.

The overarching goal is to restore scapular morphology as closely as possible to physiologic values. Although the morphologic reference values described above are currently used to guide planning and procedure selection, they have not been independently validated as definitive operative thresholds. Further clinical studies are needed to determine their predictive value and to refine the indications for each corrective procedure.

## Surgical technique

### Overview

The procedure includes 4 major steps when an isolated PACO is performed (Fig. 4):

1. Exposure
2. Guided posterior acromial osteotomy
3. Reduction and fixation of the acromial osteotomy
4. Deltoid reattachment and closure

**Figure 4:**
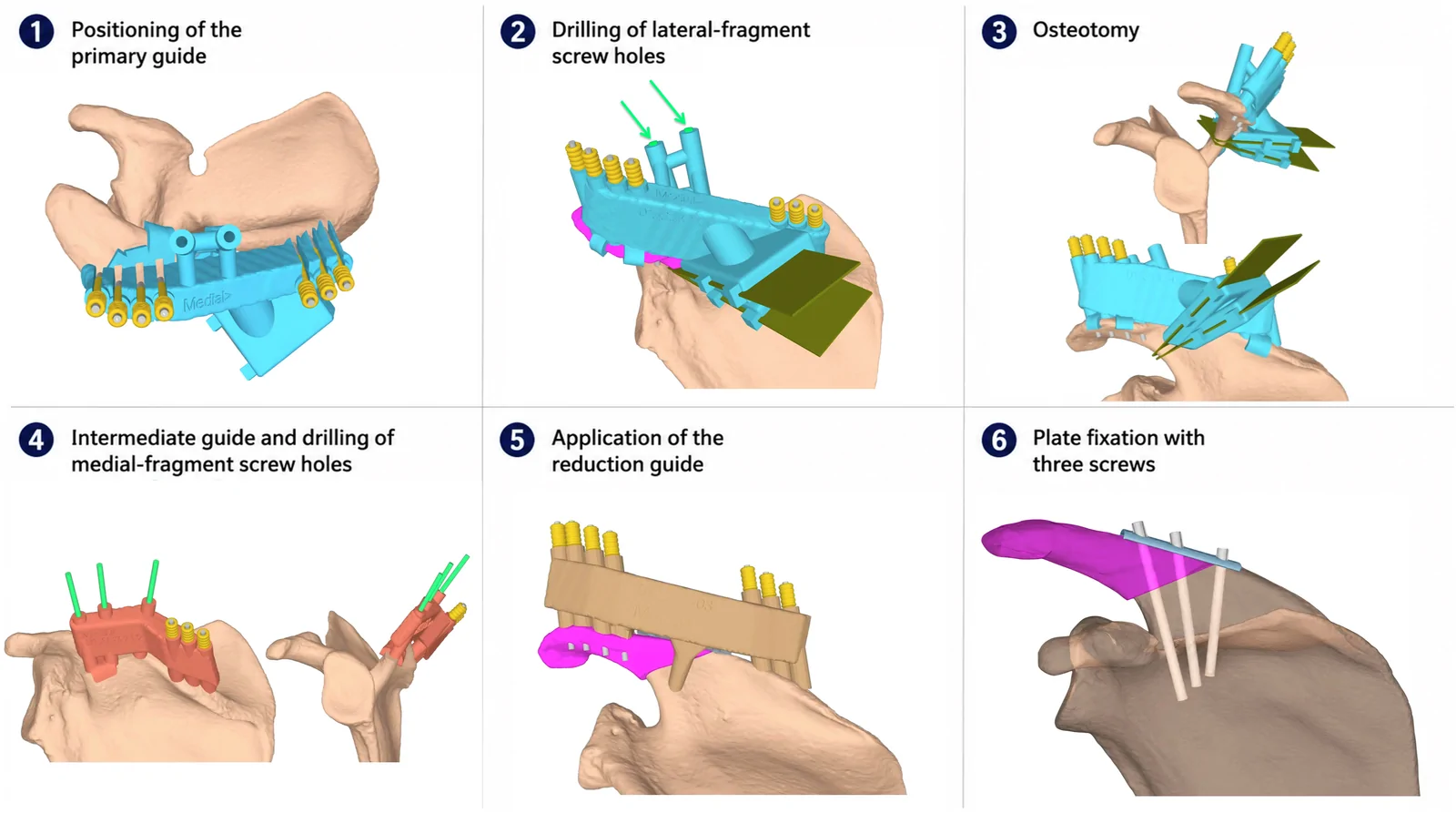
Patient-specific guide sequence for posterior acromial corrective osteotomy (PACO). (1) Positioning and fixation of the primary cutting guide with seven K-wires. (2) Drilling of the two gliding holes in the lateral acromial fragment. (3) Patient-specific wedge osteotomy through the superior and inferior cutting slots. (4) Placement of the intermediate guide and drilling of three pilot holes in the medial fragment. (5) Application of the reduction guide to reproduce and maintain the planned correction. (6) Final fixation with a 3-hole one-third tubular plate and three cortical screws.

When an additional posterior glenoid opening-wedge osteotomy is performed, the technique includes 3 additional steps:

3. Posterior glenoid osteotomy
4. Reduction and fixation of the acromial osteotomy
5. Harvest of the glenoid wedge graft
6. Fixation of the glenoid osteotomy
7. Deltoid reattachment and closure

### Exposure

All procedures are performed under general anesthesia, with regional anesthesia (interscalene block) used for post-operative analgesia according to institutional practice. The patient is positioned in the lateral decubitus position, with the arm draped free, and supported in a sterile mechanical arm holder.

The entire shoulder and upper extremity are prepared and draped. The ipsilateral iliac crest is also prepared and draped when a concomitant posterior glenoid osteotomy is planned and iliac crest autograft may be required.

A posterior L-shaped skin incision is made along the superior border of the scapular spine, extending laterally to the mid-lateral acromion. At this point, the incision is extended distally in line with the deltoid fibers. Skin flaps are elevated to expose the scapular spine and the posterior–middle deltoid raphe.

The deltoid is split bluntly approximately at the raphe. The exact location and length of the incision are determined by the footprint of the first cutting guide, which must be seated securely on the posterolateral acromion without interference from the deltotrapezial fascia after deltoid release. The posterior deltoid is detached from the posterolateral acromion and scapular spine. Tag sutures are placed in the posterolateral deltoid to facilitate mobilization and later repair (Fig. 5).

**Figure 5:**
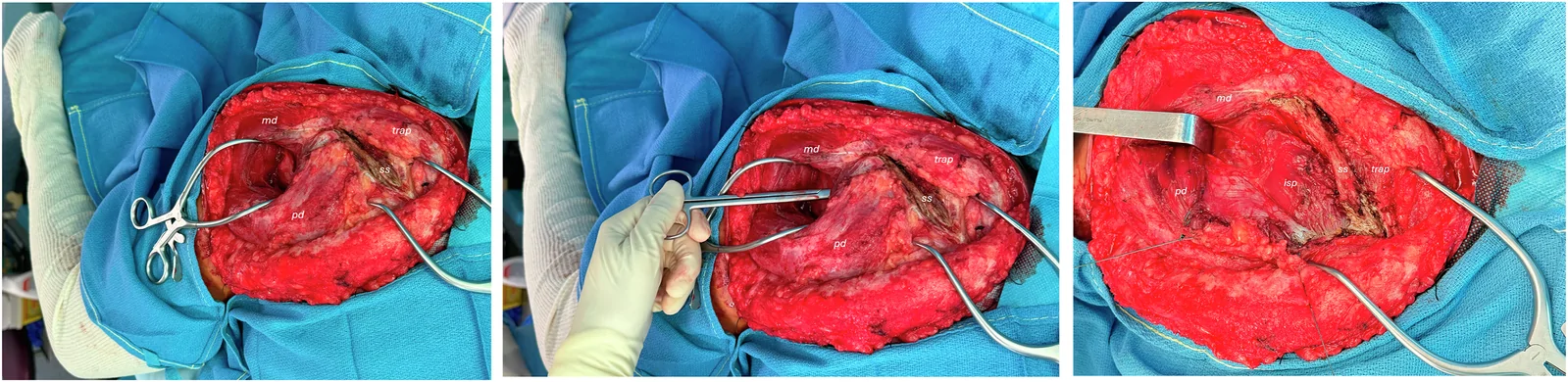
Exposure of the posterior acromion. Intraoperative photographs of a left shoulder. **(Left)** Skin flaps are elevated to expose the scapular spine and the posterior–middle deltoid raphe. The deltoid is then bluntly split along the raphe to develop the interval between the middle and posterior deltoid. **(Center)** The posterior deltoid is carefully elevated from the scapular spine while protecting the underlying infraspinatus muscle with scissors to avoid inadvertent injury during detachment. **(Right)** The posterior deltoid is detached from the posterolateral acromion and scapular spine. Tag sutures are placed in the posterolateral deltoid to facilitate mobilization and subsequent repair. *md*, middle deltoid; *pd*, posterior deltoid; *isp*, infraspinatus; *ss*, scapular spine; *trap*, trapezius.

### Guided posterior acromial osteotomy

The scapular spine and posterior acromion are thoroughly cleared of soft tissues using electrocautery and periosteal elevation to obtain a perfectly clean bony surface and allow accurate guide seating.

Patient-specific 3D-printed models and guides (MyOsteotomy system, Medacta) are used to confirm precise guide positions on the posterior acromion and scapular spine. After verifying and testing proper seating on the 3D-printed scapular model, the cutting guide is applied to the patient until the same stable “clip-on” fit is obtained. Guide stability is verified by gentle toggling in multiple directions; in particular, medial translation of the guide must not be possible (Fig. 6). Accurate guide seating is critical to reproduce the planned correction.

**Figure 6:**
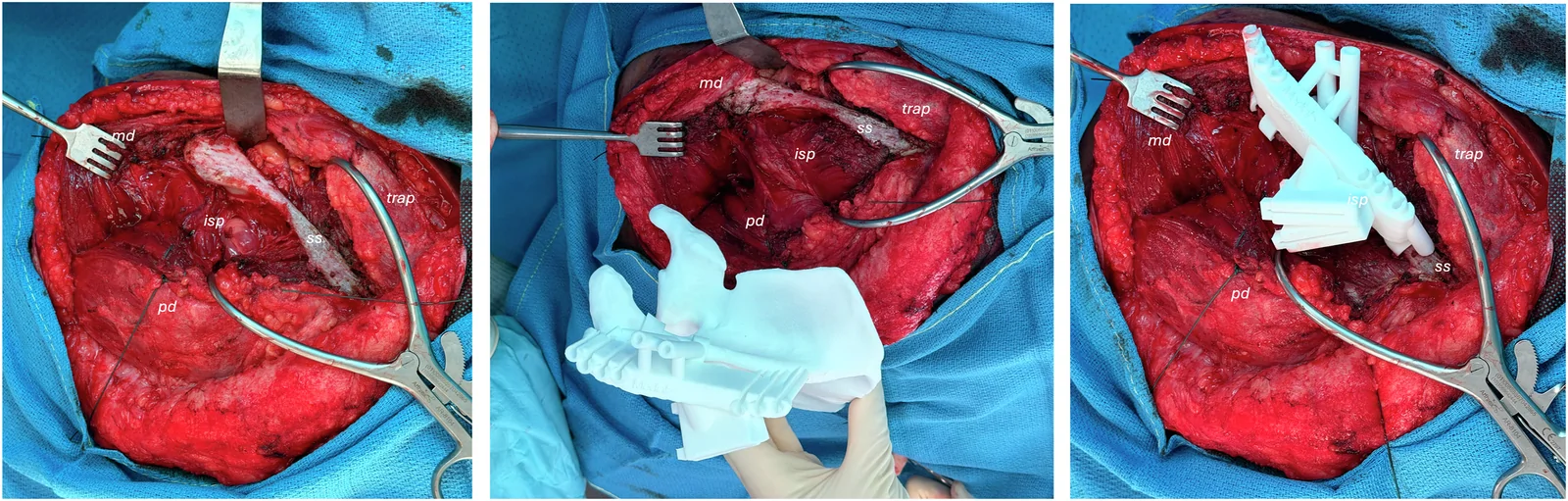
Positioning of the patient-specific cutting guide. Intraoperative photographs of a left shoulder. **(Left)** The posterior acromion and scapular spine are cleared of residual soft tissues to obtain a clean bony surface. **(Center)** Guide positioning and seating are first checked on the patient-specific 3-dimensional printed scapular model (MyOsteotomy system; Medacta, Castel San Pietro, Switzerland). **(Right)** The cutting guide is then applied to the patient until a stable “clip-on” fit is obtained, with no medial translation on gentle testing. *md*, middle deltoid; *pd*, posterior deltoid; *isp*, infraspinatus; *ss*, scapular spine; *trap*, trapezius.

The guide is secured with 3 medial and 4 lateral 2.5-mm terminally threaded Kirschner wires. Fixation is initiated with the lateral wires and then completed with the medial wires. After stable fixation of the guide, 2 3.5-mm gliding holes for ultimate screw fixation of the acromion to the spine are drilled through the guide using drill sleeves/limiters to avoid unintended penetration into the medial fragment (Fig. 7).

**Figure 7:**
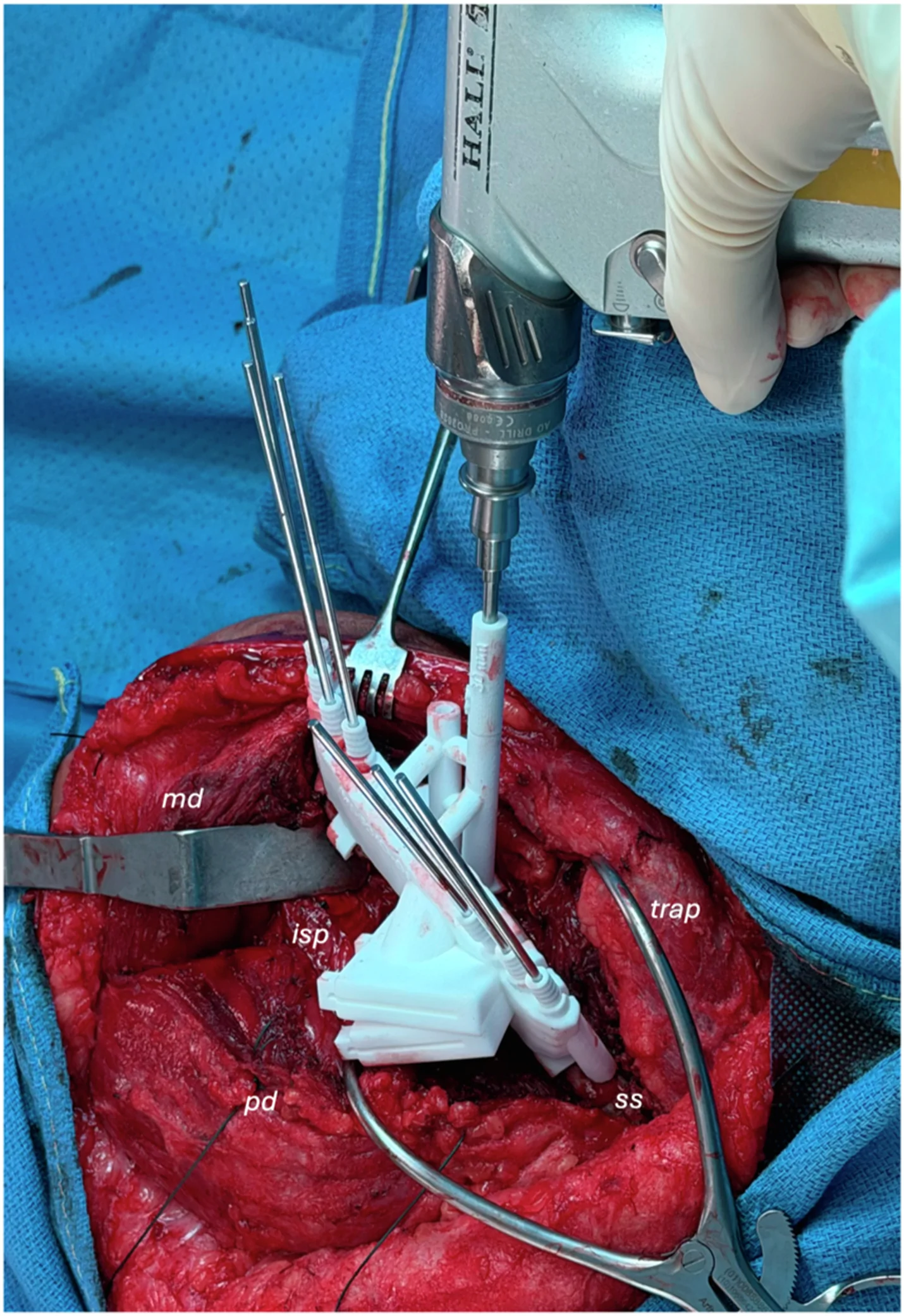
Drilling of the gliding holes. Intraoperative photograph of a left shoulder. After stable fixation of the patient-specific guide, two 3.5-mm gliding holes for subsequent screw fixation of the acromion to the scapular spine are drilled through dedicated sleeves with depth limiters to prevent inadvertent penetration into the medial fragment. *md*, middle deltoid; *pd*, posterior deltoid; *isp*, infraspinatus; *ss*, scapular spine; *trap*, trapezius.

A patient-specific posterior acromial wedge osteotomy is then performed using a 0.9 mm oscillating saw blade, and the wedge is removed. The guide has 2 cutting slots (superior and inferior), and the inferior cut is performed first (Fig. 8). The excised wedge can be compared with a 3D-printed model of the planned wedge to confirm that the osteotomy matches the pre-operative plan (Figs. 8 and 9).

**Figure 8:**
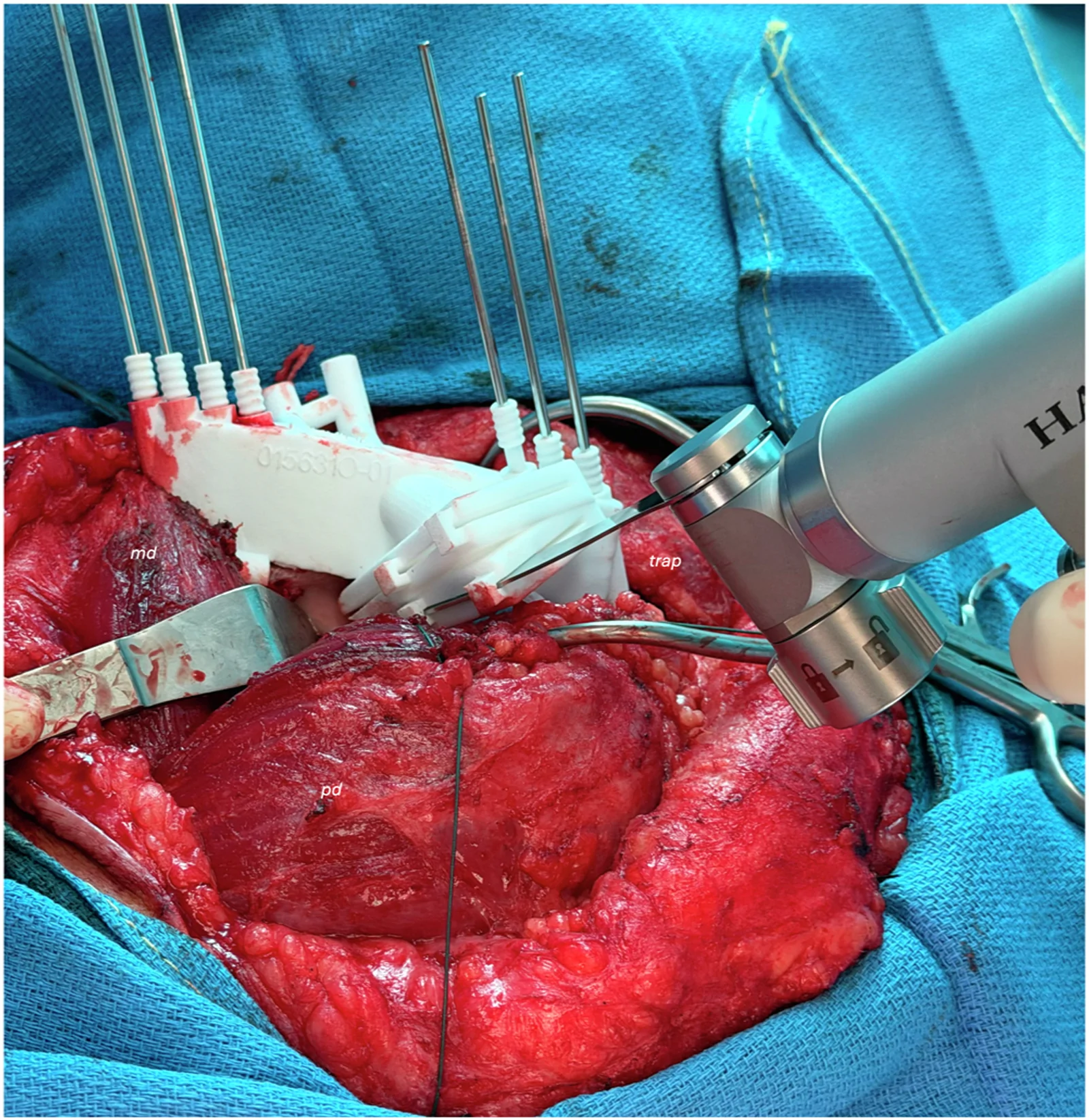
Posterior acromial wedge osteotomy. Intraoperative photography of a right shoulder. A thin oscillating saw blade is used through the guide cutting slots to create the planned wedge osteotomy; the inferior cut is performed first, followed by completion of the wedge and removal of the bone segment. *md*, middle deltoid; *pd*, posterior deltoid; *trap*, trapezius.

**Figure 9:**
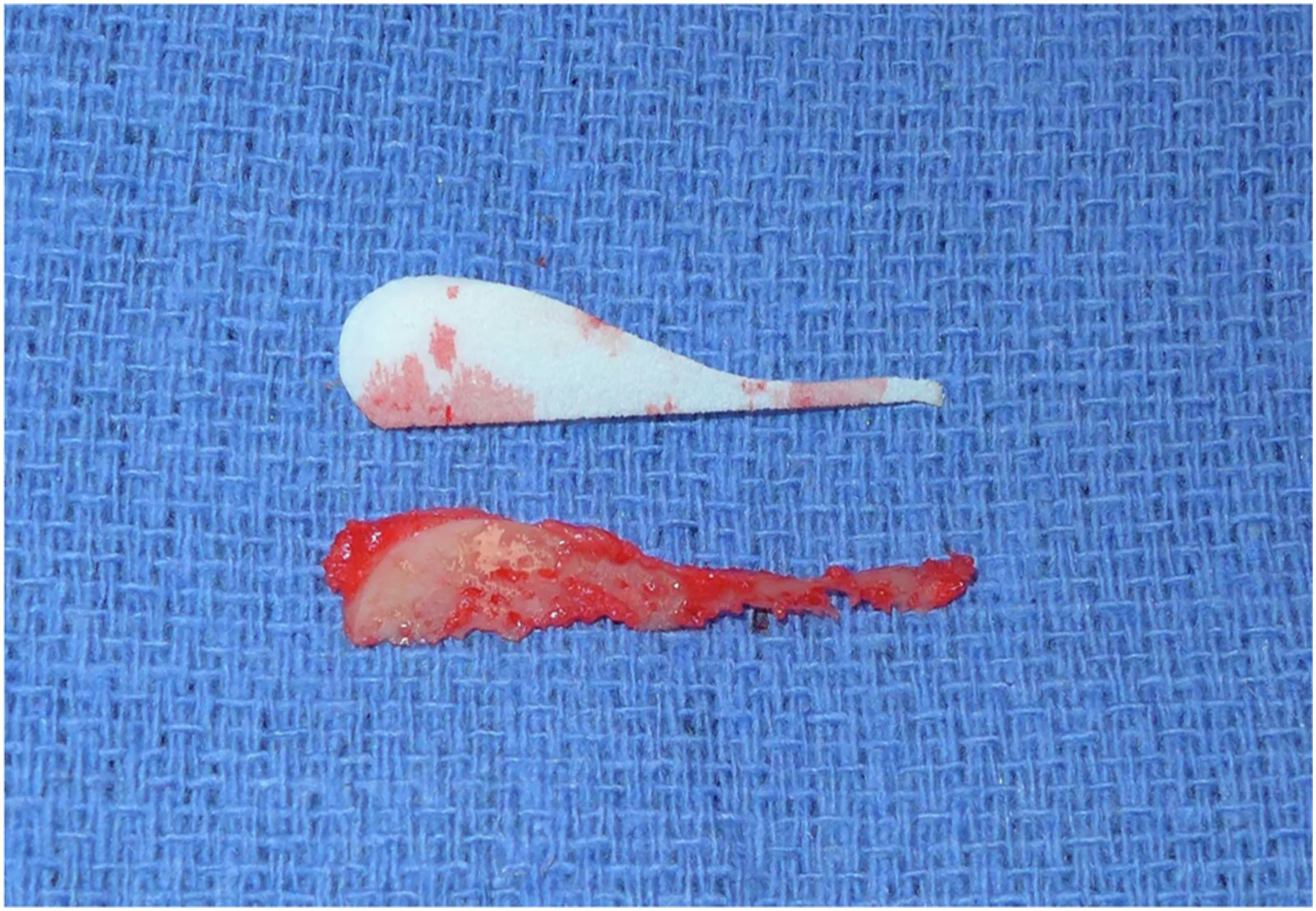
Posterior acromial wedge osteotomy. Intraoperative photography. The excised wedge can be compared with a three-dimensional-printed model of the planned wedge to confirm that the osteotomy matches the pre-operative plan.

The cutting guide is removed while the K-wires are left in place. The lateral acromial fragment is mobilized with a Kocher clamp, avoiding excessive manipulation of the lateral Kirschner wires within the fragment. The acromioclavicular joint is exposed from inferiorly and is completely released by dividing the acromioclavicular capsule while preserving the coracoclavicular ligaments (Fig. 10). This release is essential to allow sufficient mobilization of the lateral fragment to achieve the planned reduction. From this inferior approach, the coracoacromial ligament often also requires release to allow posterior translation of the acromial fragment and facilitate anatomic reduction.

**Figure 10:**
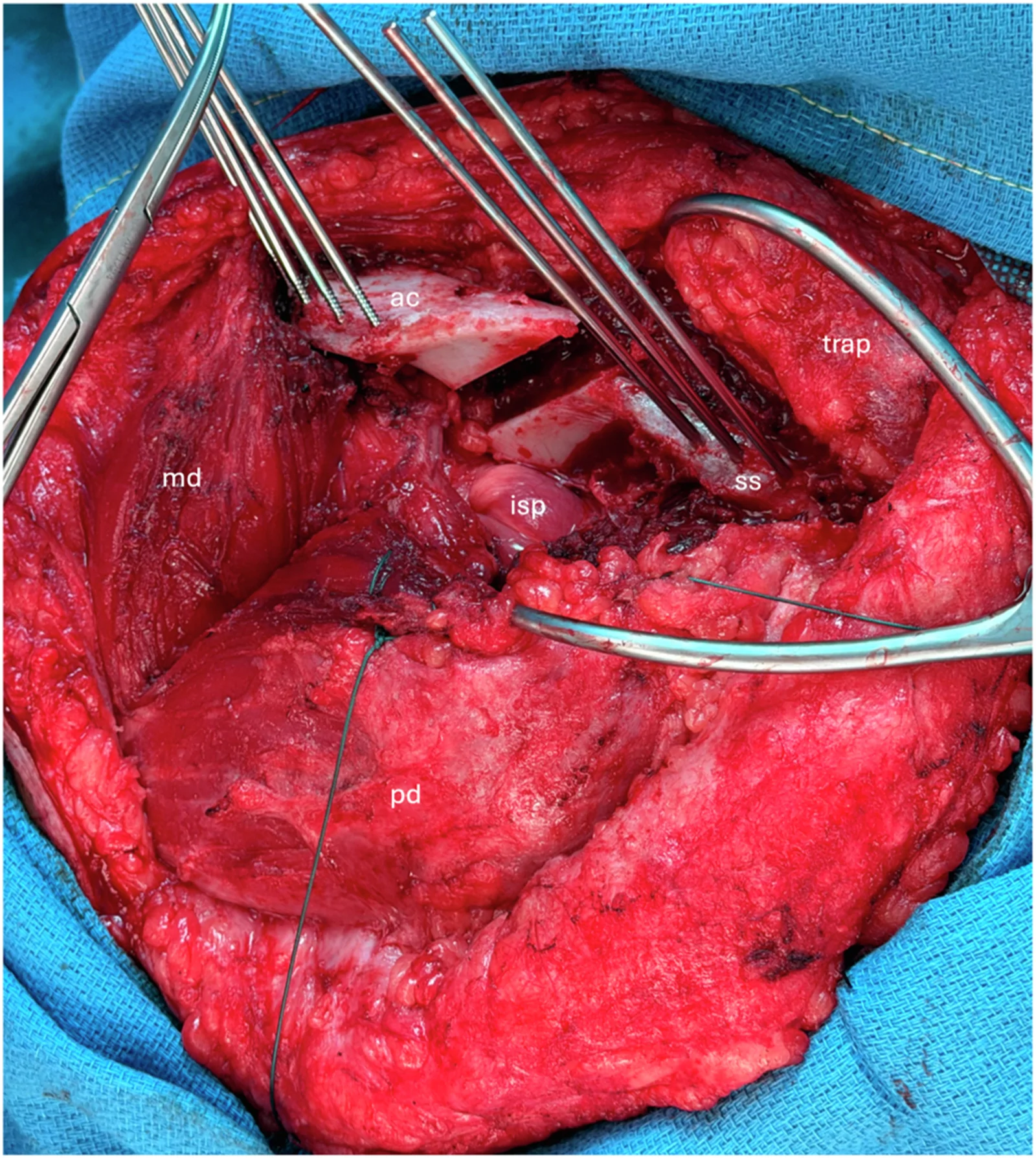
Posterior acromial wedge osteotomy. Intraoperative photograph of a left shoulder. After completion of the osteotomy, the cutting guide is removed while the Kirschner wires are left in place. The two osteotomy fragments are shown separated, exposing the osteotomy surfaces. *ac*, acromion; *md*, middle deltoid; *pd*, posterior deltoid; *isp*, infraspinatus; *ss*, scapular spine; *trap*, trapezius.

An intermediate 3D-printed guide is then mounted on the 3 medial K-wires and applied to the scapular spine at the osteotomy site to guide the entry point and trajectory of the 2.8 mm pilot holes, thereby facilitating accurate placement of the final 3.5-mm screws and compression across the osteotomy (Fig. 11).

**Figure 11:**
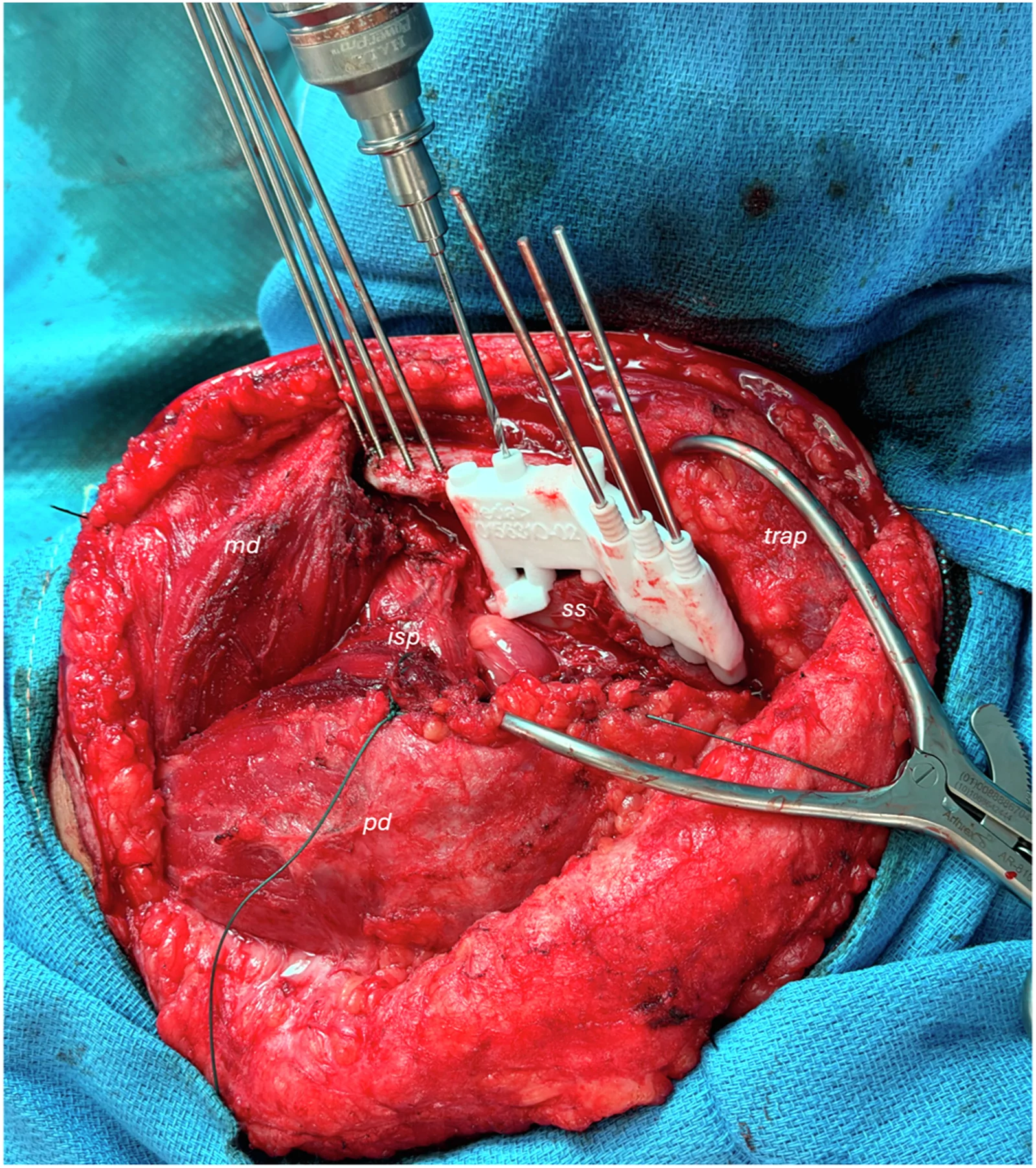
Preparation of the medial screw trajectories. Intraoperative photograph of a left shoulder. An intermediate patient-specific guide is positioned on the medial Kirschner wires to guide drilling of the pilot holes in the scapular spine for subsequent screw fixation. *md*, middle deltoid; *pd*, posterior deltoid; *isp*, infraspinatus; *ss*, scapular spine; *trap*, trapezius.

### Posterior glenoid osteotomy (when performed)

With the acromial osteotomy completed and the acromial fragment mobilized, a blunt interval is developed between the infraspinatus and teres minor, and a blunt Gelpi retractor is placed to expose the posterior capsule. A small Hohmann retractor is positioned at the inferior aspect of the posterolateral aspect of the scapular neck to improve visualization. A T-shaped capsulotomy is performed, and each capsular limb is tagged with absorbable sutures for later repair.

A Fukuda retractor is placed intra-articularly. The posterior labrum is inspected, then divided and elevated superiorly and inferiorly to expose the posterior glenoid wall; both labral limbs are tagged for later repair.

Bone exposure proceeds from lateral to medial with care taken to protect the suprascapular nerve. In our experience, posterior glenoid exposure is facilitated by performing the acromial osteotomy first, as mobilization of the lateral acromial fragment improves visualization.

The planned osteotomy line is confirmed using the patient-specific 3D model. A blade osteotome is introduced parallel to the joint line, at least 1.5 cm from the articular surface to create the posterior glenoid osteotomy while preserving the anterior cortex as a hinge. The osteotomy is gradually opened without fracturing the anterior cortex. In some cases, correction of marked inferior tilt is also required. In that setting, controlled completion of the osteotomy through the anterior hinge at its inferior aspect may be necessary to allow the planned reorientation. A patient-specific 3D-printed wedge spacer corresponding to the planned correction is impacted into the osteotomy to maintain the planned opening during the remainder of the procedure (Fig. 12).

**Figure 12:**
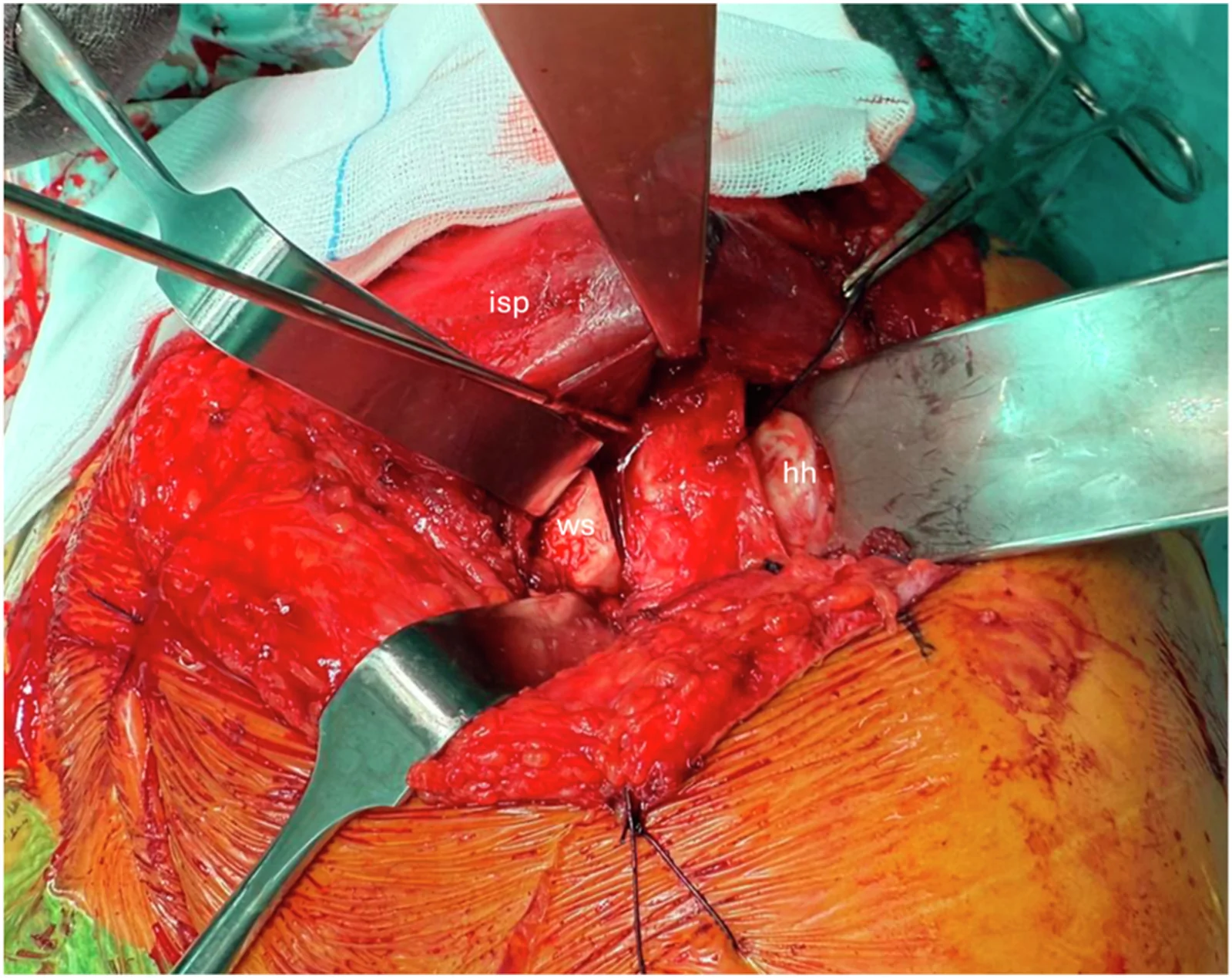
Posterior glenoid corrective osteotomy (GCO). Intraoperative view of a right shoulder. A split is created between the infraspinatus (isp) and teres minor. A Fukuda retractor is placed intra-articularly to retract the humeral head (hh). A patient-specific three-dimensional-printed wedge spacer (ws) corresponding to the planned correction is impacted into the osteotomy to maintain the planned opening during the remainder of the procedure.

### Acromial reduction and fixation

Attention is returned to the acromial osteotomy. Two 2.0-mm K-wires are placed through the corresponding predrilled screw trajectories to guide reduction and provide provisional fixation. A third, more medial hole is predrilled for subsequent fixation of a 3-hole one-third tubular plate (see below). A 3D-printed reduction guide is applied over the original 7 K-wires to reproduce the planned correction and maintain fragment position. The achieved reduction is compared with the reduction on the corresponding 3D-printed model (Fig. 13).

**Figure 13:**
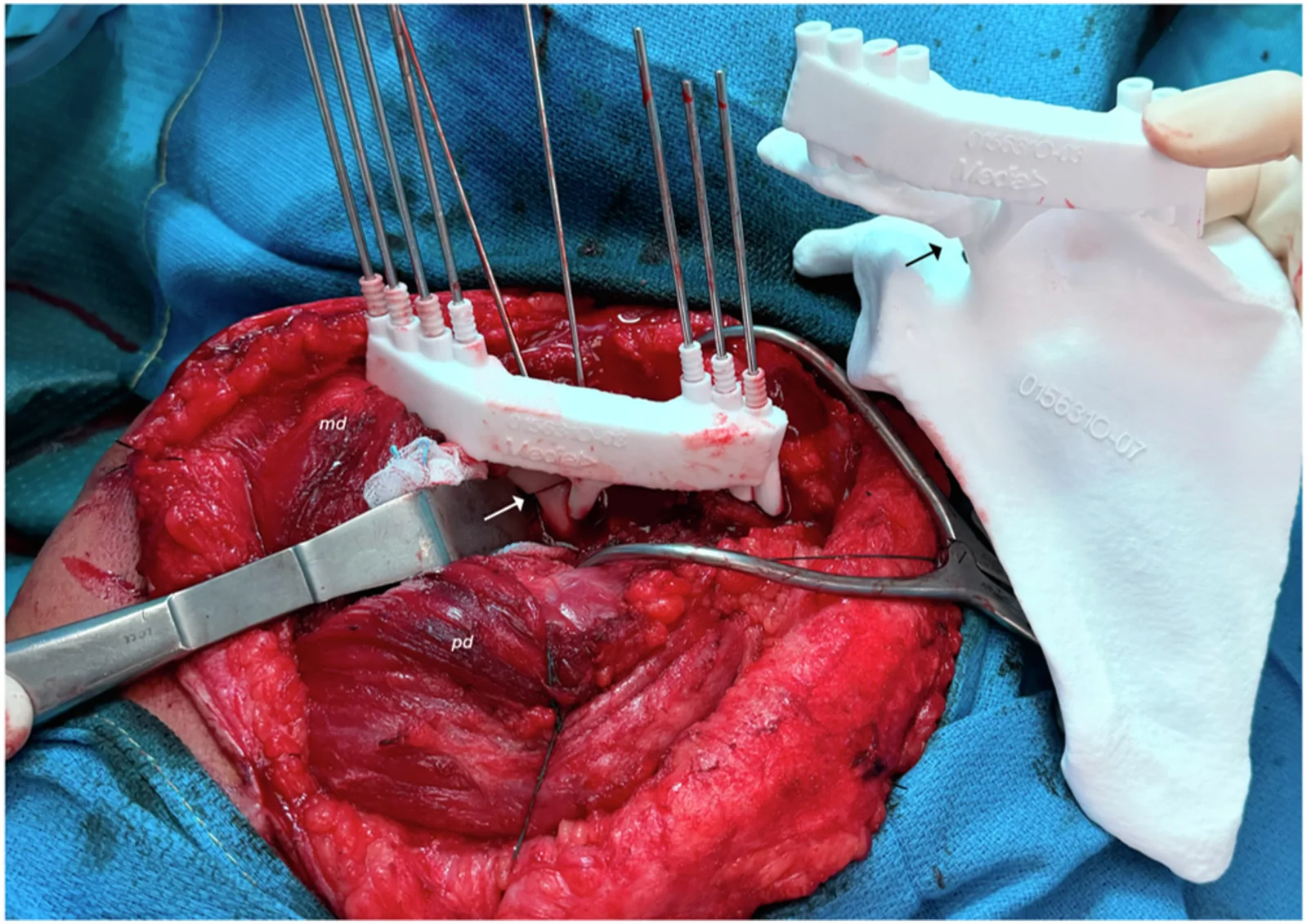
Patient-specific reduction guide for posterior acromial corrective osteotomy (PACO). Intraoperative photograph of a left shoulder. After provisional reduction with Kirschner wires placed through the predrilled screw trajectories, the patient-specific reduction guide is applied to reproduce and maintain the planned correction. The white arrow indicates the reduced osteotomy site, while the black arrow shows the corresponding correction on the 3-dimensional printed scapular model, demonstrating concordance between the planned and achieved reduction. *md*, middle deltoid; *pd*, posterior deltoid.

A 3-hole 3.5-mm one-third tubular plate (Bonebridge, Inc., Switzerland) is applied across the osteotomy and secured with 3 3.5-mm cortical screws (Bonebridge, Inc., Switzerland) of appropriate length. Two screws engage both fragments to generate compression across the osteotomy, and the most medial screw secures the plate within the medial fragment. The plate functions as a washer, distributing load under the screw heads to reduce the risk of screw-head penetration into the acromial fragment and subsequent pull-through. In the authors' experience, using a one-third tubular plate as a washer is important to achieve consistent compression and maintain reduction during healing; insufficient compression or secondary displacement may increase the risk of nonunion. The reduction guide and screw entry sites are planned with the dimensions of the one-third tubular plate in mind; if alternative fixation hardware is used, the pre-operative plan should be adjusted accordingly to ensure plate–screw compatibility and intended compression. The provisional Kirschner wires are then removed (Fig. 14).

**Figure 14:**
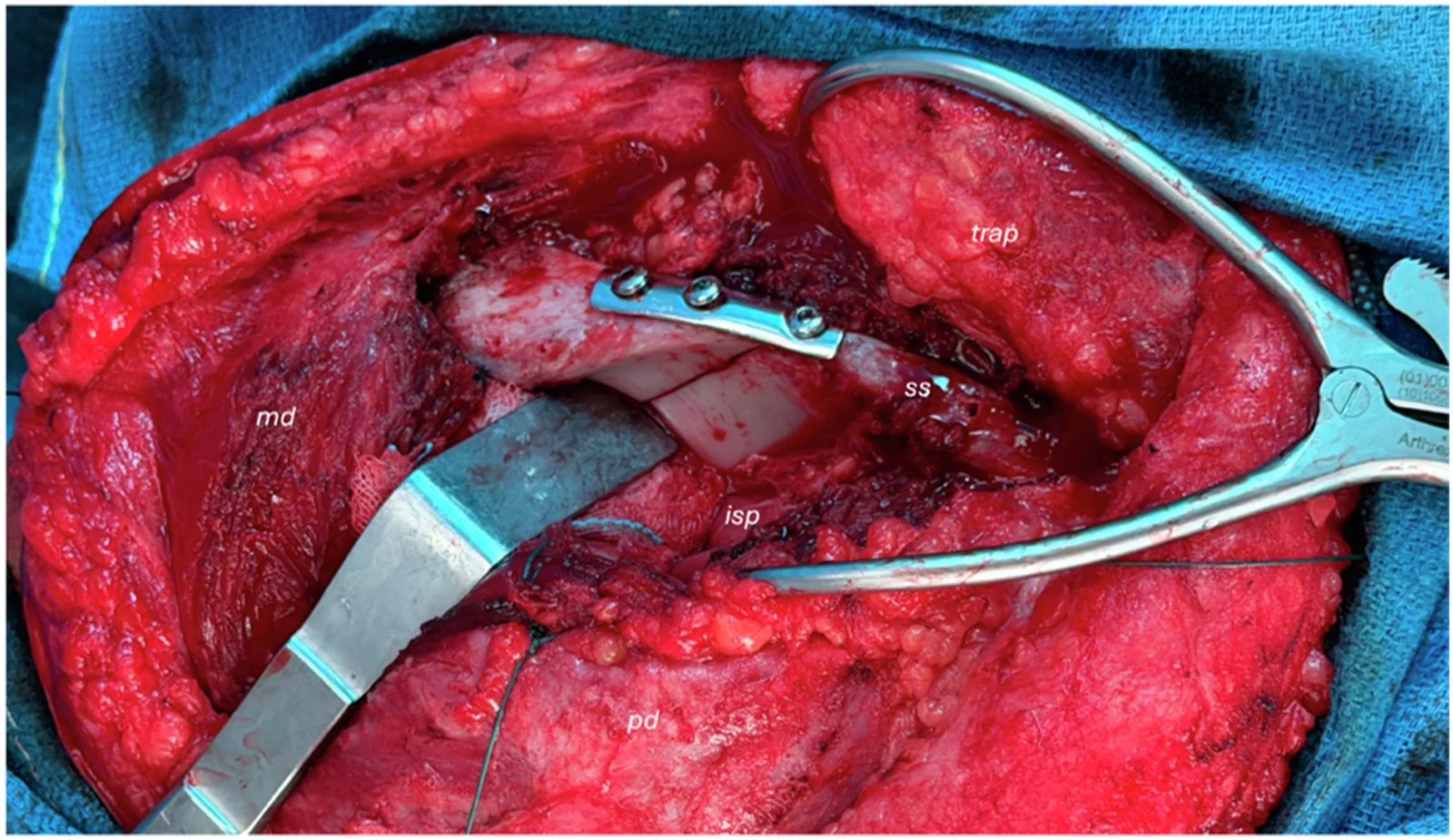
Final fixation of the osteotomy. Intraoperative photograph of a left shoulder. The corrected acromial fragment is stabilized to the scapular spine with a 3-hole one-third tubular plate and three 3.5-mm cortical screws, providing compression across the osteotomy. *md*, middle deltoid; *pd*, posterior deltoid; *isp*, infraspinatus; *ss*, scapular spine; *trap*, trapezius.

### Harvest of the glenoid wedge graft (when performed)

An autograft wedge is harvested in the planned shape to correct glenoid version and inclination using a cutting guide provided with the guide set. Potential donor sites include the medial scapular spine, distal clavicle, or iliac crest. Because the scapular spine is already exposed, it may be preferentially used; however, iliac crest or clavicular cortical bone may provide a more rigid graft to help maintain correction over time.

The patient-specific cutting guide is fixed to the donor site with 2 1.4-mm K-wires, and the graft is harvested with an oscillating saw through the guide. The guide is removed, while the K-wires may be left temporarily to facilitate graft manipulation.

### Fixation of the glenoid osteotomy (when performed)

The posterior glenoid osteotomy site is re-exposed with the Gelpi retractor in the infraspinatus–teres minor interval, the Fukuda retractor intra-articularly, and the inferior Hohmann retractor beneath the glenoid neck.

The 3D-printed wedge spacer is removed and replaced with the harvested autograft, which is oriented as planned and press-fit into the osteotomy. The posterior labrum and posterior capsule are repaired with absorbable sutures, and the retractors are removed. If the osteotomy opening is large or the graft appears unstable, supplemental fixation can be added using a 2.7 L-shaped plate to stabilize the graft and osteotomized fragment.

### Deltoid reattachment and closure

The lateral 4 K-wire holes and the 2 medial K-wire entry points are completed from posterior to anterior with a 2.5-mm drill bit to create transosseous tunnels for deltoid reattachment. The first reattachment suture is placed through the most lateral and anterior tunnel to restore the anatomical position and tension of the posterior deltoid. Six to 7 nonabsorbable braided sutures are then passed from the posterior scapular spine and posterior acromion and through the posterior deltoid and tied using sliding knots (Nice Knots,^5^ cow hitch knots^29^) or secured with Mason-Allen stitches, according to surgeon preference (Fig. 15). The posterior deltoid is then repaired to the middle deltoid with a running absorbable suture. The deltotrapezius fascia is closed with heavy absorbable loop suture, covering the plate, followed by routine subcutaneous and skin closure.

**Figure 15:**
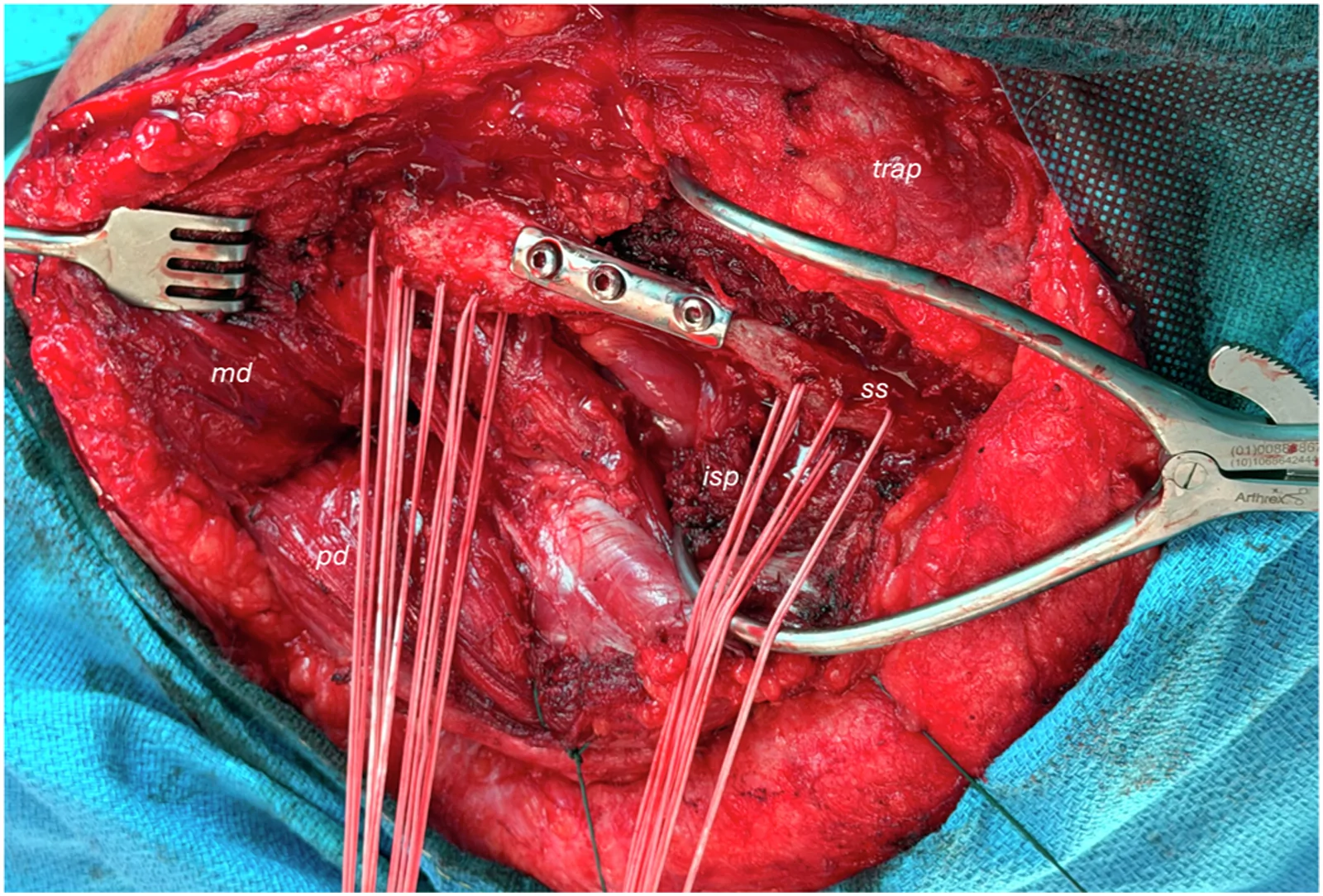
Posterior deltoid reattachment. Intraoperative photograph of a left shoulder. The seven pre-existing Kirschner wire holes are used as transosseous tunnels for the passage of seven nonabsorbable sutures through the posterior acromion, scapular spine, and posterior deltoid before final repair. *md*, middle deltoid; *pd*, posterior deltoid; *isp*, infraspinatus; *ss*, scapular spine; *trap*, trapezius.

### Post-operative immobilization and rehabilitation

Patients are immobilized in an abduction pillow for 4 to 6 weeks post-operatively. When both the acromion and glenoid are corrected, immobilization for 6 weeks is preferred. Early motion of the elbow, wrist, and hand is encouraged. Active-assisted shoulder range of motion is initiated at 4 to 6 weeks and progressed until full passive motion is restored. At approximately 3 months, radiographs and CT are obtained to assess healing of the osteotomies; strengthening is initiated thereafter. Return to unrestricted physical activities is typically allowed at 6 months, provided that healing is confirmed.

## Discussion

Scapular morphology, and particularly posterior acromial morphology, appears to contribute to the pathomechanism of posterior shoulder instability.^2^^,^^3^^,^^11^, ^12^, ^13^, ^14^^,^^20^ While current strategies range from soft tissue stabilization to glenoid-based osseous procedures, they do not directly address posterior acromial insufficiency in patients in whom it is a major contributor to instability, contributing to persistent or recurrent symptoms.^18^

A recent cadaveric biomechanical study using an advanced active-motion shoulder simulator showed that isolated posterior capsulolabral injury had only a limited effect on posterior humeral head translation, whereas glenoid and acromial malalignment produced substantially greater alterations in glenohumeral kinematics.^12^ Similarly, isolated correction of glenoid retroversion or posterior acromial deficiency does not fully restore biomechanics as long as other morphologic deviations of the scapula persist.^12^ These findings support the need for a comprehensive 3-dimensional assessment of scapular morphology to guide an anatomically based reconstructive strategy. This rationale underlies the SCOPE approach, which aims to restore scapular morphology toward physiologic values by addressing the patient-specific combination of deformities during a single reconstruction.^9^^,^^10^

For correction of posterior acromial deformity, 2 primary procedures may be considered: PABB^32^^,^^33^ and PACO. In rare cases, the 2 may be combined. Although PABB may be technically simpler and less invasive, it may be suboptimal in cases with insufficient lateral acromial extension, in which an added posterior bone block may remain too medial relative to the humeral head to provide adequate posterior restraint. Therefore, in shoulders with a short and high posterior acromion, restoration of normal anatomy with a PABB may not be possible. In addition, limited lateral acromial extension is commonly associated with a low CSA, which may be associated with altered glenohumeral loading.^23^^,^^39^ In such cases, PACO may be preferred because it allows realignment of the posterior acromion and can simultaneously address posterior height, posterior coverage, and lateral extension, whereas PABB is primarily an augmentative procedure. Whether a more complete restoration of posterior acromial morphology with PACO results in improved midrange kinematics compared with PABB, which may primarily provide restraint at end-range elevation or abduction, remains unknown.

The SCOPE approach relies on patient-specific 3-dimensional pre-operative planning, including comparison of the pathologic scapula with a mean SSM of healthy shoulders to quantify deformity and to plan individualized correction. In the presence of increased glenoid retroversion and inferior tilt, a posterior GCO may be added.

This global reconstruction concept is intended to improve stability by providing posterior osseous restraint to posterior translation^12^, ^13^, ^14^ and may favorably modify glenohumeral loading by restoring a more physiologic CSA.^39^ Early clinical results have been encouraging.^9^^,^^10^

During the development and refinement of this surgical technique, potential complications included undercorrection or overcorrection, nonunion, hardware irritation, and suprascapular nerve injury. Accordingly, the technique is presented after an iterative refinement process and substantial experience (>80 cases) by, or under the direct supervision of, the senior author, with the goal of standardizing the steps, improving reproducibility, and minimizing these complications. Meticulous step-by-step execution remains essential.

Larger clinical series are needed to define optimal indications, clinical outcomes, and their relationship to the accuracy of correction, durability of clinical benefit, complication rates, and the potential impact on osteoarthritis progression. Moreover, clinically meaningful morphologic thresholds that should trigger scapular correction and specifically the selection of GCO, PABB, and/or PACO remain to be established. The practical morphologic reference values proposed in this article represent the authors' current planning framework and should not be interpreted as definitive standalone operative thresholds. Larger clinical series are required to validate their predictive value and to further refine the indications for scapular correction and the selection of GCO, PABB, PACO, or combined procedures.

Overall, the available clinical observations,^25^, ^26^, ^27^^,^ biomechanical studies,^28^, ^29^, ^30^, ^31^ and early clinical results^32^^,^^33^ emphasize the importance of 3-dimensional scapular assessment when selecting the most appropriate treatment for posterior instability.

## Conclusion

The SCOPE approach relies on patient-specific 3-dimensional CT-based planning to quantify posterior scapular deformity and to select the appropriate corrective procedure. PACO is a guided realignment technique designed to restore PAH, posterior coverage, and lateral extension; when indicated, it can be combined with a posterior GCO to address severe glenoid retroversion. This article provides a reproducible step-by-step description of exposure, guide positioning, osteotomy execution, reduction, fixation, and deltoid reattachment and highlights technical pearls and pitfalls intended to improve accuracy and minimize complications. Further clinical studies are needed to establish morphologic thresholds for procedure selection and to evaluate healing, complications, and durability of correction.

## Disclaimers:

Funding: No funding was disclosed by the authors.

Conflicts of interest: Jean-David Werthel“ receives royalties for shoulder arthroplasty implants from Stryker. Matthias Zumstein receives grants from Medacta; is a designer for VIMS, Zimmer, and Bonebridge; and receives non-financial support from Angiocrine Biosciences, unrelated to the submitted work. “Any additional authors, their immediate families, and any research foundations with which they are affiliated have not received any financial payments or other benefits from any commercial entity related to the subject of this article.
